# Repeatability and Agreement of Macular Thickness Measurements Obtained with Two Different Scan Modes of the Optovue RTVue Optical Coherence Tomography Device

**DOI:** 10.4274/tjo.galenos.2018.88972

**Published:** 2019-04-30

**Authors:** Mehmet Kola, Mehmet Önal, Adem Türk, Hidayet Erdöl

**Affiliations:** 1 Karadeniz Technical University Faculty of Medicine, Department of Ophthalmology, Trabzon, Turkey

**Keywords:** Eye, optical coherence tomography, retina

## Abstract

**Objectives:**

To evaluate the repeatability and agreement of macular thickness measurements obtained with E-MM5 and MM6, two different scan modes, on the Optovue RTVue optic coherence tomography (OCT) device.

**Materials and Methods:**

Three consecutive macular thickness measurements in 30 healthy volunteers were taken using the OCT device E-MM5 and MM6 scan modes. The repeatability and agreement of these measurements obtained from the two scan modes and divided into nine anatomical regions based on early treatment diabetic retinopathy study were subjected to statistical analysis.

**Results:**

The mean age of the participants was 29.7±6.39 years. Intraclass correlation (all ICC values ≥0.86) and coefficient of variation (all coefficient of variation values ≤2%) analyses of consecutive OCT measurements in the nine regions of the macula obtained in both E-MM5 and MM6 scan modes gave high repeatability rates. Mean macular thickness values in the foveal region were 243.76±21.79 μm in E-MM5 mode and 247.04±19.83 μm in MM6 mode (p=0.543). Values for measurements obtained in E-MM5 and MM6 scan modes in parafoveal macular regions were also statistically similar (p>0.05 for all). However, a statistically significant difference was observed between the two modes in perifoveal macular measurements, except in the superior region.

**Conclusion:**

The Optovue RTVue OCT device gives highly repeatable measurement results for macular thicknesses in both E-MM5 and MM6 scan modes. However, it should be considered that measurements performed in E-MM5 and MM6 modes give different results in perifoveal regions.

## Introduction

With advances in optical coherence tomography (OCT) technology, images of the ocular tissues can now be acquired in high resolution. OCT examinations provide useful information for the diagnosis of numerous ocular disorders and enable more detailed follow-up and more sensitive evaluation of treatment response.^1,2,3,4,5,6^ As a result, OCT is used extensively in the diagnosis and treatment of many eye disorders.^7,8,9,10,11,12^ This has led to the need for more sensitive and reproducible OCT measurements.

There are currently various OCT devices being produced by many different manufacturers.^13^ These devices use different algorithms, and are reported to give different measurement results.^14,15^ Of these, the Optovue RTVue is a spectral domain OCT device that uses 830 nm light to acquire 26000 A-scans per second for image resolution of 5 μm. Two different scanning modes of the device, E-MM5 and MM6, enable the acquisition of fovea-centered macular images.^16^ Comparing these two modes and determining their repeatability in patients with whom they are used for macular evaluation may result in more accurate diagnosis and treatment. However, there are few studies on this subject in the literature. Therefore, this study was performed to evaluate the repeatability and agreement of macular thickness measurements obtained using two different retinal scan modes, E-MM5 and MM6, of the Optovue RTVue OCT device.

## Materials and Methods

Ethics committee approval was obtained prior to this cross-sectional study, and all participants were informed about the study and provided informed consent. Volunteers aged 9-44 years with no systemic diseases or ocular disorders other than refractive error were included in the study.

All participants underwent a detailed ophthalmological examination including autorefractometry, best corrected visual acuity and intraocular pressure measurement, and slit-lamp examination of the anterior and posterior segments. Participants whose eye examination revealed ocular pathologies other than refractive error (strabismus, nystagmus, ptosis, corneal opacity, uveitis, cataracts, maculopathy, glaucoma, etc.), refractive error greater than ±4 D spherical equivalent, cup/disc ratio ≥0.4 or asymmetry ≥0.2 between the cup/disc ratios of both eyes, or intraocular pressure >21 mmHg were excluded from the study. In addition, participants with a history of ocular surgery or trauma and those who were unwilling to participate or uncooperative during the examination were also excluded. Individuals with signs of systemic disease were not included.

The Optovue RTVue (RT100 software version 6.3, Optovue Inc., Fremont, CA, USA) spectral-domain OCT device was utilized in this study. For each participant, fovea-centered macular measurements were acquired in 3 consecutive scans using both of the device’s preprogrammed scanning modes: E-MM5 (0.9 s; outer 6 x 6 mm grid of 13 horizontal and 13 vertical lines with 668 A-scans each and inner 4 x 4 mm grid of 8 horizontal and 8 vertical lines with 400 A-scans each) and MM6 (0.27 s; 12 radial scans with 1024 A-scans each in a circular area 6 mm in diameter).^16^ All measurements were performed in the same session within a period of 10 minutes, with participants remaining seated at the OCT device and resting by lifting their heads from the device. The device’s internal fixation system was used to prevent eye movements during OCT measurements and the participants’ pupils were not dilated before the scan. All measurements were performed by the same researcher experienced in performing OCT.

Criteria used to ensure reliable OCT image acquisition in the study were that the images had no artifacts, were properly centered, clearly showed distinct retinal layers, and had signal strength index (a scan quality indicator) greater than 50.

**Figure 1:**
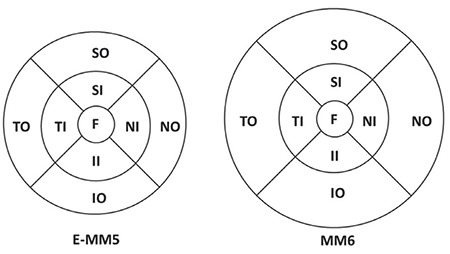
The early treatment diabetic retinopathy study grid divides optic coherence tomography measurements into nine anatomic zones: Central fovea (F), nasal inner (NI), temporal inner (TI), inferior inner (II), superior inner (SI), nasal outer (NO), temporal outer (TO), superior outer (SO), inferior outer (IO) macula. The central ring is 1 mm in diameter, the inner ring is 3 mm in diameter, and the diameter of the outer ring is 5 mm in E-MM5 scan mode and 6 mm in MM6 scan mode

OCT measurements of the macular area were divided into nine anatomical regions according to ETDRS (Early Treatment Diabetic Retinopathy Study) (Figure 1).^17^ The inner and outer macula are delineated by rings 3 mm and 5 mm in diameter in E-MM5 mode and 3 mm and 6 mm in diameter in MM6 mode, respectively. In both scan modes, the central 1 mm diameter ring represents the fovea.

SPSS 13.0.1 (SPSS, Chicago, IL, USA; license no: 9069728, KTU, Trabzon, Turkey) software was used for statistical analyses. Numerical data were presented as mean ± standard deviation. The one-sample Kolmogorov–Smirnov test was used to analyze whether the numerical data were normally distributed. Data from the participants’ right eyes were used in the analysis of OCT measurements. Repeated measures were compared using paired-samples t test. Agreement between measurements was assessed using intraclass correlation (ICC) test and coefficient of variation (CV) values. CV was calculated as the percentage of the ratio of the standard deviation to the mean ([standard deviation/mean] x 100). A CV <10% was considered high repeatability, and CV <5% was considered very high repeatability. ICC values of 0-0.2 were accepted as very poor repeatability, 0.21-0.4 as poor repeatability, 0.41-0.6 as moderate repeatability, 0.61-0.8 as good repeatability, and ≥0.81 as excellent repeatability. A p value ≤0.05 was considered statistically significant.

## Results

The study included a total of 30 patients, 18 females (60%) and 12 males (40%), with a mean age of 29.7±6.39 (19-44) years. For all participants, best corrected visual acuity was 20/20, intraocular pressure was normotonic, and findings in slit-lamp anterior and posterior segment examination were within normal limits. OCT measurement results obtained from the participants’ right eyes using E-MM5 and MM6 scan modes are shown in Tables 1 and 2. The ICC values of all measurements made in both modes indicated excellent repeatability (ICC>0.81 for all).

Tables 3 and 4 show the CV values for comparisons of consecutive measurements performed in each scan mode. The CV values obtained using both modes also indicated very high repeatability (CV ≤2% for all).

The mean values of consecutive measurements obtained using the E-MM5 and MM6 scan modes are presented in Table 5. Central and paracentral macular measurements were similar between the two modes, while perifoveal macular measurements showed significant differences in all but the superior quadrant.

## Discussion

The repeatability of a diagnostic tool is very important for making an accurate diagnosis. Repeatability of retinal thickness measurements is critical in the follow-up of progression or treatment in retinal diseases. In this study, we evaluated the repeatability of OCT measurements of the macula obtained in two different scan modes and the agreement between them.

There are various studies in the literature analyzing the repeatability of OCT measurements. Even with older generation time-domain OCT devices, the ICC values of macular thickness measurements demonstrated excellent repeatability.^18,19^ In a study using Fourier-domain OCT in pediatric patients, Altemir et al.^20^ reported CV and ICC values of 0.97% and 0.942 for macular thickness measurement and 1% and 0.94 for macular volume measurement, respectively. Therefore, the repeatability of consecutive OCT measurements in the macular area is known to be very high. Similarly, in our study, the ICC and CV values of repeated OCT measurements supported the reliability of the results.

When the macular measurements obtained using E-MM5 mode were evaluated according to the ETDRS map, those in the temporal inner macula had the lowest ICC while those in the superior outer macula had the highest ICC. Similarly, CV values of measurements acquired in E-MM5 mode were lowest for superior outer thickness and perifoveal macular volume and highest for foveal thickness and volume measurements.

With MM6 mode, foveal thickness and volume measurements had the lowest ICC, while nasal inner macular thickness and perifoveal macular volume measurements had the highest ICC. CV values for MM6 measurements were lowest for perifoveal macular volume measurements and highest for inferior inner macular and temporal outer macular thickness measurements.

In a study by Garcia-Martin et al.^13^ using a different Fourier-domain OCT device than the one used in our study, the lowest CV for repeated measures was in the nasal inner macula (0.6%), while the highest was in the foveal and inferior outer macula (1.8%). In the same study, measurements of the nasal inner macula had the highest ICC values (0.992), while the lowest ICC was in the superior outer macular area (0.832). In another Fourier-domain OCT study, Menke et al.^21^ reported that the CV values of all macular thickness measurements obtained according to the ETDRS map varied between 0.38% and 0.86%, with the lowest CV observed in the outer temporal macula and the highest in the inner temporal macula.^21^ In a comparative study by Pinilla et al.^6^ using two different Fourier-domain OCT devices, it was reported that the CV values for repeated mean macular thickness measurements of the devices were between 2.2-2.95% and all ICC values were over 0.919. The authors also reported differences in the measurements obtained using the two different OCT devices.^6^ In their study, measurements obtained according to the ETDRS map in healthy eyes using the Cirrus OCT device showed the lowest CV in the nasal outer macula (0.7%; ICC=0.963) and the highest CV in the inferior inner macula (3.4%; ICC=0.92). In measurements of healthy eyes obtained using a Spectralis OCT device, the lowest CV was in the inferior inner macula (0.3%; ICC=0.996) and the highest CV was in the temporal outer macula (1.3%; ICC=0.927).^6^

As the studies mentioned above suggest, the different scanning algorithms in different OCT devices can cause various deviations in the repeatability values of OCT measurements made according to the ETDRS map. Moreover, in some studies, these discrepancies may have been due in part to measuring different retinal areas or having multiple operators performing OCT measurements. Thus, a direct comparison of studies in the literature is not possible. Nevertheless, it is still clear that macular thickness measurements performed using OCT devices have satisfactory repeatability.

## Conclusion

This study evaluated the repeatability of measurements obtained using the E-MM5 and MM6 scan modes of the Optovue RTVue OCT device in healthy individuals, and compared the agreement between them. The CV and ICC values for repeated measures were similar to those reported in other studies in the literature. Macular thickness measurements performed using both E-MM5 and MM6 scan modes of the Optovue RTVue OCT device yielded results with highly repeatability. This could make an important contribution to patient follow-up. However, it must be kept in mind that perifoveal measurements obtained using E-MM5 and MM6 modes yielded different results. This is due to the different software algorithms of the scan modes. In E-MM5, the outer macula is shown in a 3-5 mm zone within a scan area 5 mm in diameter, while in MM6 the outer macula is shown in a zone between 3-6 mm in a scan area 6 mm in diameter. Therefore, consistently using the same retinal scan mode throughout a patient’s follow-up is the best approach. Furthermore, Schneider et al.^22^ reported that radial scans were superior when evaluating small macular holes. Thus, the MM6 scan mode, which has a radial scanning protocol, may be more useful than E-MM5 scan mode in the evaluation of these macular pathologies.

## Figures and Tables

**Table 1:**
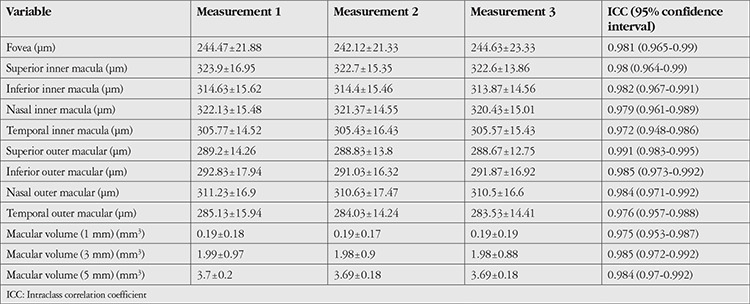
The mean (± standard deviation) and intraclass correlation coefficient values of three consecutive measurements of the participants’ right eyes (n=30) using the E-MM5 scanning mode of the optical coherence tomography device

**Table 2:**
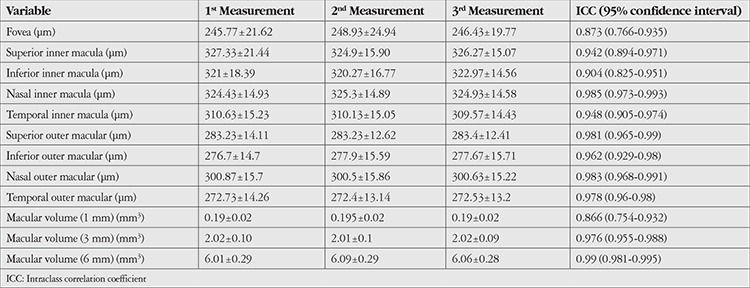
The mean (± standard deviation) and intraclass correlation coefficient values of three consecutive measurements of the participants’ right eyes (n=30) using the MM6 scanning mode of the optical coherence tomography device

**Table 3:**
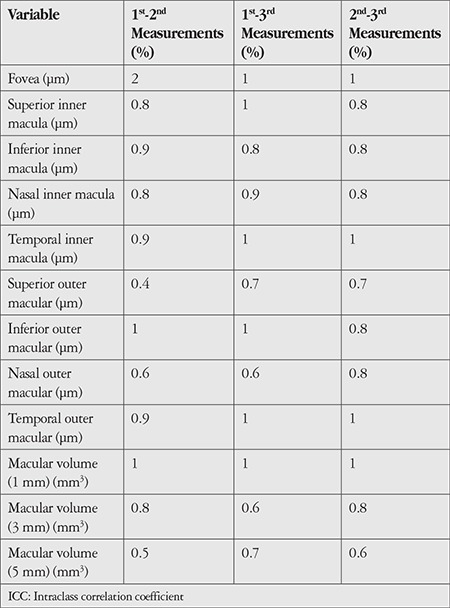
Coefficients of variation for pairwise comparisons of three consecutive measurements of the participants’ right eyes (n=30) using the E-MM5 scanning mode of the optical coherence tomography device

**Table 4:**
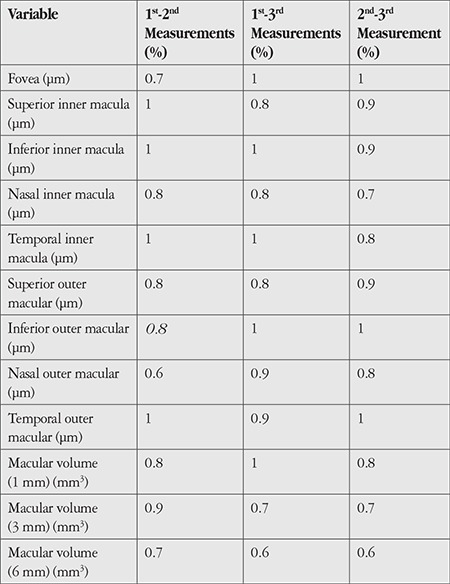
Coefficients of variation for pairwise comparisons of three consecutive measurements of the participants’ right eyes (n=30) using the MM6 scanning mode of the optical coherence tomography device

**Table 5:**
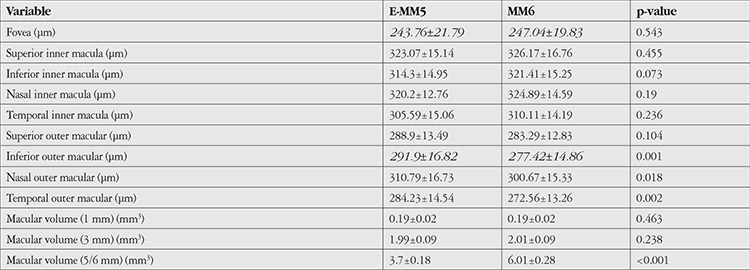
Comparisons of mean measurements obtained from the participants’ right eyes (n=30) using the E-MM5 and MM6 scanning modes of the optical coherence tomography device
